# A multimodal understanding of the role of sound and music in gendered toy marketing

**DOI:** 10.1371/journal.pone.0311876

**Published:** 2024-11-06

**Authors:** Luca Marinelli, Petra Lucht, Charalampos Saitis

**Affiliations:** 1 Centre for Digital Music, Queen Mary University of London, London, United Kingdom; 2 Center for Interdisciplinary Women’s and Gender Studies, Technical University of Berlin, Berlin, Germany; Kitami Institute of Technology, JAPAN

## Abstract

Literature in music theory and psychology shows that, even in isolation, musical sounds can reliably encode gender-loaded messages. Musical material can be imbued with many ideological dimensions and gender is just one of them. Nonetheless, studies of the gendering of music within multimodal communicative events are sparse and lack an encompassing theoretical framework. The present study attempts to address this literature gap by employing a critical quantitative analysis of music in gendered toy marketing, which integrated a content analytical approach with multimodal affective and music-focused perceptual responses. Ratings were collected on a set of 606 commercials spanning a ten-year time frame and strong gender polarization was observed in nearly all of the collected variables. Gendered music styles in toy commercials exhibit synergistic design choices, as music in masculine-targeted adverts was substantially more abrasive—louder, more inharmonious, and more distorted—than in feminine-targeted ones. Thus, toy advertising music appeared deliberately and consistently in line with traditional gender norms. In addition, music perceptual scales and voice-related content analytical variables explain quite well the heavily polarized affective ratings. This study presents a empirical understanding of the gendering of music as constructed within multimodal discourse, reiterating the importance of the sociocultural underpinnings of music cognition. We provided a public repository with all code and data necessary to reproduce the results of this study on *github.com/marinelliluca/music-role-gender-marketing*.

## 1 Introduction

The advertising industry is one of the most influential agents in the (re)shaping of new and existing meaning systems. According to Jhally [1], advertising seems to be obsessed with gender and sexuality, as these are arguably the most used social resources in the industry. There are several reasons for this, firstly because gender is easily identifiable and accessible, gender segments are also easily measurable and, more importantly, they are large and profitable [2]. Advertisers use color, shape, texture, packaging, logos, verbiage, graphics, product names, and sound (and music) to define the gender of a brand [3]. The marketing of toys in particular is notoriously stereotyped by gender, which has been under increased critical scrutiny in recent years in public and academic debate [4].

In an analysis of the ethics and science of this debate, Fine and Rush [4] came to the conclusion that gendered toy marketing is neither ethical, nor scientifically sound, nor even financially advantageous for businesses. Being based on gender essentialist perspectives that are “ludicrously old-fashioned and offensively out of touch”, gendered toy marketing misrepresents the much more flexible interests of children. Even Liz Truss, at that time United Kingdom’s Education Minister, critiqued the practice of producing and advertising gender-specific toys in an interview with the Telegraph [5]. Sweet [6] even argued that toys appeared to be more divided by gender in 2014 than they were fifty years before. Such public backlash specifically has prompted businesses, governments, and policymakers to start steering away from gendered toy marketing. Following a 2017 UK report on gender stereotypes in advertising [7], the Committee of Advertising Practice (CAP) and the Broadcast Committee of Advertising Practice (BCAP) introduced a rule in the UK Advertising Codes prohibiting harmful gender stereotypes in advertising, effective from 14 June 2019. A 12-month review confirmed the rule’s effectiveness [8], leading to its retention with added clarification that would initially offer guidance only for cases involving sexualization, objectification, and body image. This highlighted the need for further monitoring of areas where guidance was insufficient, particularly in relation to ads targeting children and other vulnerable groups. Whereas in 2022, Spain has explicitly banned gender stereotypes in toy advertising to “promote and encourage a plural, egalitarian and stereotype-free image of minors” [9]. After commissioning a study that surveyed nearly 7000 parents and children from around the globe, the LEGO Group [10], the largest toy maker in the world, re-evaluated its position and vowed to remove gender biases from its products. Despite such initiatives, gender-based market segmentation is still a predominant practice in the global toy industry [11, 12]. Although the grassroot research by Let Toys Be Toys—https://www.lettoysbetoys.org.uk/tvads2021—has been showing progress in the UK market since 2015, stereotypes remained widespread. A possible explanation as to why this is still common practice is that the industry could be trapped in a vicious circle. When children’s interests are, to some considerable extent, prescribed by toy companies, then the same manufactured desires can shape the development of future toys, thereby giving the impression that toy companies are providing their customers with what they want [13].

Gender polarization in TV advertising aimed at children has been consistently found in a large body of studies spanning 40 years [11, 14–22]. Differences between commercials targeted at girls, boys, and mixed audiences have been found in terms of sound (voices, background music and sound effects), language, transitions, and camera work, setting, interactions and activities, and colors. Particularly in terms of sound and music, Verna [14] found no female narrators in the advertisements targeted at boys or at the mixed audience that she analyzed. And, at that time, male voice-overs predominated over female ones even in commercials targeted at girls. Welch et al. [15] noted an improvement, since female narrators were at least predominant in commercials targeted at girls, concluding that in general the sex of the voice-over matched the target audience of the commercials. But again, they found that male narrating voices occurred more often in mixed audience commercials. Subsequent research confirmed the same trend with little variation (as reported in Mitra et al. [21]). A few studies analyzed, besides the sex of the narrator, other auditory characteristics. Welch et al. [15] found that commercials targeted at boys had more noise, louder music, and more sound effects. Lewin-Jones and Mitra [23] conversely found that the music used in girls’ advertisements is generally softer and more likely to have a sung narration style. Whereas, Johnson and Young [19] identified what they called “gender exaggeration” in voice-overs. Male voice-overs were often exaggeratedly deep, growl-like, or aggressive, whereas female voice-overs were often very high-pitched and singsong.

In terms of the effects that such marketing strategies have on the viewers, a recent UK report by the Fawcett Society [24] showed that gender stereotypes in early childhood are at the root of problems with body image, limited perceived career choices, eating disorders, record male suicide rates as well as violence against women. According to social cognitive theory and cultivation theory [25, 26], repeated exposure to gender-stereotyped messages can influence behaviors, beliefs, and attitudes. In particular, media can influence emotional socialization [27], and gender differences in emotion expression might emerge. In this regard, little work has been done to examine the audience’s emotional response to toy commercials [28, 29]. Furthermore, to the best of our knowledge, no previous research has attempted to study differences in the *intended* emotional impact of gendered toy commercials, that is, the emotional profile that marketers purposefully aim to evoke in their audience. Taking into account that music is found in more than 90% of television adverts [30], and interpreting it as an inherently multimodal discourse, a critical analysis of gender markers in children’s TV adverts can help investigate the relationship between music and hegemonic discourses on gender, and to probe further research towards a commercial and contemporary musical semiotics of gender. In addition, analyzing music and sound in gendered advertising targeted at children allows a privileged glance into the birthplace of *music-primed gender schemas* (see sec. 2.3).

### 1.1 Framing gender in musical practice

Gendered meanings encoded in music are the central focus of the analysis of this study. Therefore, it makes sense to make a formal distinction between sex and gender. The former describes biological differences that affect reproductive, physiological, and metabolic systems, and which categorize individuals into female, male, and intersex. Instead, according to the seminal work of Butler [31], gender is a socially constructed binary that defines and differentiates ‘men’ and ‘women’ as the only two intelligible categories of people. For Butler, it is the (mandatorily) heterosexual desire that binds masculinity and femininity in a binary, hierarchical relationship. It should be specified that biological and sociocultural dimensions cannot always be precisely separated, as some of the social expectations for both masculinity and femininity are shaped by biological facts, for example, that women can usually become pregnant or that men have a propensity to have larger and stronger bodies.

According to Schiebinger et al. [32], gender exists on a spectrum defined by three sociocultural dimensions—*identity, relational, and normative*—that throughout history have shaped behaviors, products, technologies, environments, and knowledge, for individuals and in society at large. *Gender identities* refer to “how individuals or groups perceive and present themselves in relation to gender norms”. These identities are context-specific and can overlap with other facets such as ethnicity and cultural heritage. *Gender relations*, instead, involve the ways individuals interact with others and institutions based on their sex and gender identity. These relations influence social interactions in various settings and become evident in power dynamics, such as between male patients and female physicians. Finally, *gender norms* are created, and propagated through various social platforms, including institutions like families and schools, interactions among individuals, and cultural products like films, literature, and TV commercials. These norms set expectations and attitudes about behaviors and roles appropriate for different genders, influencing even the fields of science and technology. They often uphold gender stereotypes and are perpetuated through unequal resources distribution and discrimination in various societal sectors. Gender norms are not static, instead, they evolve, varying across different historical periods, cultures, and locations, and can differ in diverse social contexts.

It is in terms of pervasive gender norms that we can understand the gendering of musical practices. Discussions on the masculine and feminine in musical aesthetics have been documented throughout history and can be traced back to Ancient Greece and early Common Era centuries. Plato, Aristotle, Clement, Basil, and Boethius, among others, associated manly music with reason, restraint, and order, whereas music associated with women or effeminacy was thought to arouse sensuality, excitement, passion, or madness [33]. According to McClary [34, p. 17], such associations, in many historical periods, between music and ideas of sensuality and subjectivity, ultimately led to it being relegated to the feminine realm. Eventually, male musicians retaliated by prohibiting altogether female participation in music. In centuries, composers developed a musical semiotics of gender and, as these codes changed over time, studying music from this perspective could offer insights into the dominant discourses on gender at a particular time.

### 1.2 This study

**Fig 1:**
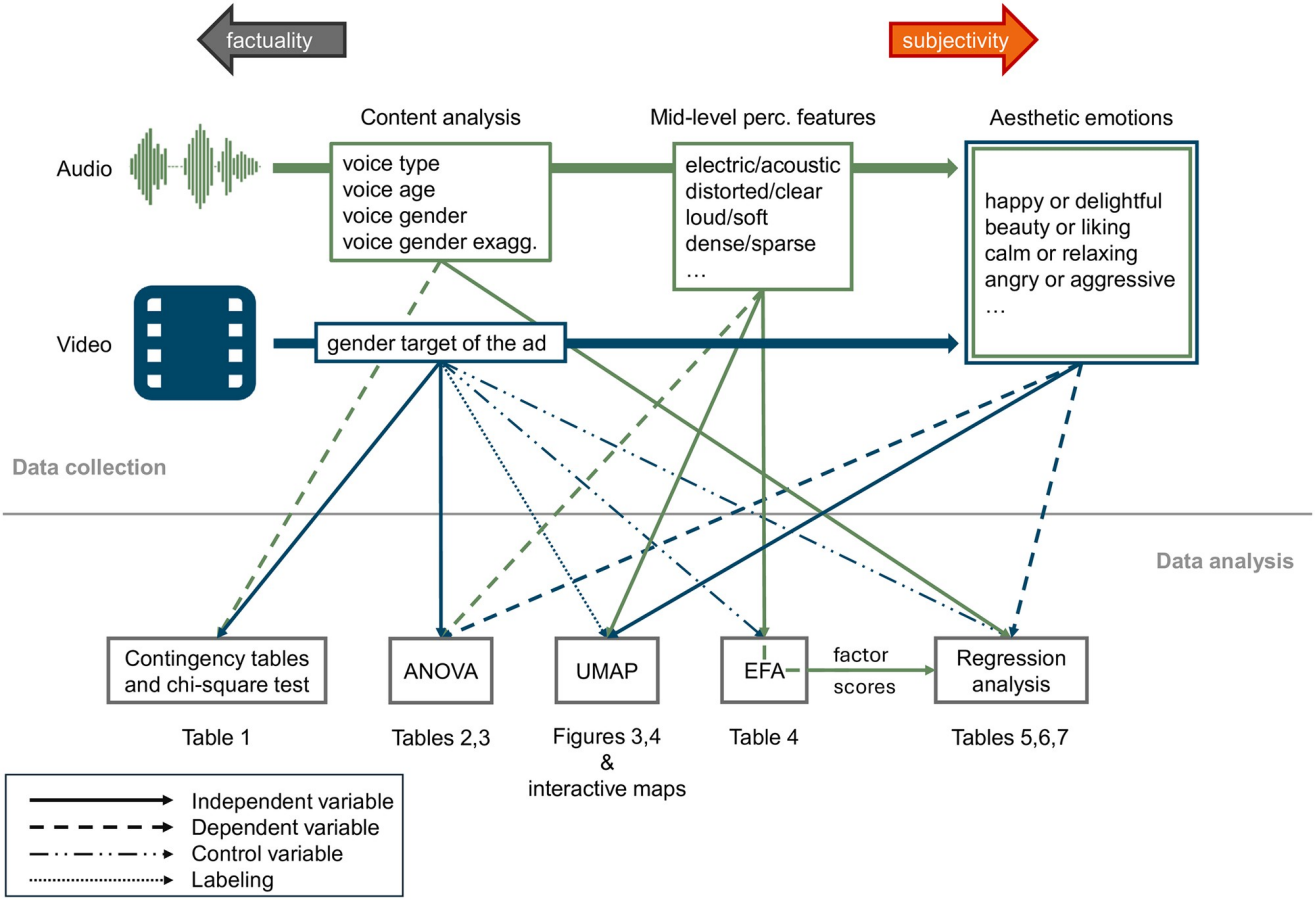
Overview of the multimodal, hierarchical data collection and of the subsequent data analysis undertaken in this study.

Our overarching research objective is to provide a basis for a theory of message production [35]. Specifically, a theory of the effects that message producers—who in the UK advertising industry are disproportionately represented by white men [36]—their decision-making or their unconscious gender biases have on the selection and composition of sound and music in gendered toy commercials. For this goal, we propose an integrative approach [37] displayed in Fig 1, which combined content analytical (CA) variables, music perceptual ratings, and multimodal affective ratings. Our framework for data collection is hierarchically organized: at a lower (more factual) level we have CA variables that were tested for reliability, and at the middle (more subjective) level are music-focused ratings, which were collected from a pool of musically trained participants. Finally, at the highest level of subjectivity we have multimodal affective ratings provided by non-experts. Inferring the gender target of toy adverts (and thus of their soundtracks) with a content analytical approach allows for a more objective standpoint from which to examine the gendering of music. We conducted our study with adults instead of collecting children’s responses. On the one hand, there exists evidence that already by age 11 children exhibit adult-like emotion recognition capabilities [38]. On the other hand, we assume the intended emotional impact to be better captured by adults, who are media savvy—or following Tagg [39] (see sec. 2.2), adults possess a more developed *store of signs* and a deeper understanding of the *sociocultural norms* shared with the message creators—and can thus best perform analysis and critique.

The following research questions and hypotheses informed the design of our study:

*RQ*1: Where are commercials from each gender target group placed, concerning each other in terms of music descriptors and affective responses? For example, according to Griffiths [40], advertisements targeted at mixed audiences consistently exhibited masculine characteristics in their formal attributes. Girls are socialized from an early age into accepting a masculinized style in media that is supposedly aimed at both genders. This appears to introduce children to a wider discourse of dominant masculinity in the media [21].
*RQ*2: To what extent does the soundtrack explain the affective responses? In other words, how relevant is the role of sound and music in conveying the affective dimensions in multimodal communicative events?

Concerning *RQ*1, we hypothesize that the soundtracks of the commercials, in terms of music perceptual descriptors will be more abrasive in masculine-targeted commercials, and more soothing in feminine-targeted ones. That is, the selection and composition of sound and music in these commercials will reflect traditional gender stereotypes (*H*1_*a*_). For example, Herget [41], and Tagg [42, 43] define masculine music as “strong,” “hard,” and “active”, and feminine music as “soft,” “tender,” and “smooth.” More specifically, Welch et al. [15] found that commercials targeted at boys had more noise, louder music, and more sound effects. In addition, the distribution of affective responses to these commercials will also reflect traditional gender stereotypes (*H*1_*b*_). Masculine-targeted commercials will be for example more aggressive and energetic than feminine-targeted ones. Regarding *RQ*2, to the best of our knowledge no previous study attempted to model affective responses to gender-based multimodal genres with respect to music perceptual features, and therefore we are unable to form hypotheses.

In summary, the main contributions of this study are:

- To approach an understanding of how the gendering of sound and music operates as a multimodal discursive process; in section 2 we proposed a series of complementary theoretical frameworks.
- A new hierarchically organized dataset, comprising 606 toy TV commercials from 2012 to 2022, CA items, music-focused, and multimodal emotion ratings, as described in section 3. To the best of our knowledge, this dataset is the largest of its kind, also covering the widest time frame.
- A quantitative analysis of the role of sound and music in gendered toy marketing, as detailed in section 4, including two interactive maps and a public repository with the code needed to reproduce the results.

## 2 Theoretical framework

In this section, we first covered different methods for analyzing gender representation in media, including content analysis, both a quantitative and qualitative method for documenting media portrayal patterns. Then we introduced Tagg’s socio-semantic perspective on extramusical meaning-making [39], emphasizing the importance of a shared cultural and ideological context for decoding intended musical messages such as gender. The concept of multimodality, popular in various academic disciplines, was also introduced to acknowledge the complexity of human communication, emphasizing the interconnectedness of different semiotic modes in meaning-making. Afterward, we moved onto how music and gender are intertwined in cognitive psychology, emphasizing that our sociocultural backgrounds shape not only musical material but also how we process and perceive music. In particular, cognitive schemas are proposed to contribute to the emergence of gendered patterns in music perception and production.

### 2.1 Studying the gender-media relation

Scholars have employed a variety of analytical tools and frameworks to uncover the nuances of gender portrayal in media. In this section, we presented a selection of key methods and theories, drawing primarily from the second chapter of *Gender and the Media* by Rosalind Gill [44].

Content analysis (CA) is used to document patterns in media. It essentially consists in counting the frequency of particular kinds of portrayal (or roles) using coding frameworks that have been agreed in advance. As such, CA can be considered both a quantitative and qualitative method, and its raw data can be easily analyzed with statistical packages. Given the relatively low costs of conducting this research, and the high-status quantitative methods have (in comparison to qualitative research), CA can be used to hold advertising agencies and broadcasters accountable for not respecting regulations or progressive agendas. For this reason, it has become the industry’s standard way of assessing gender representation. Despite this, critics have pinpointed that CA, on its own, does not distinguish between different levels of signification, as “research in the content analytic tradition tells us little about the images it examines, except how frequently they occour” (ibid, p. 43).

Discourse analysis (DA), instead, is a contested umbrella term that refers to approaches developed within a huge variety of academic disciplines. This includes, of course, disciplines that first developed models for understanding discourse, such as linguistics, social semiotics and conversation analysis. But it also refers to other approaches that apply and extend these models of understanding to their particular academic fields, such as cognitive psychology, literary criticism, and artificial intelligence [45]. It is then perhaps useful to think of DA as organized around four main themes: “a concern with discourse itself, a view of language as constructive and constructed, an emphasis upon discourse as a form of action, and a conviction in the rhetorical organization of discourse” [44, p. 58]. As for the first theme, at its most basic linguistic definition, *discourse* is anything ‘beyond the sentence’. But in our specific case, when critical theorists speak of ‘discourses on gender’ they refer to a “broad conglomeration of linguistic and non-linguistic social practices and ideological assumptions” [45] that together construct or reinforce gender power relations. The second theme is that *language is constructive*. The metaphor of construction refers to the fact that discourse is always built out of pre-existing resources, which have to be chosen among a multitude of other possibilities and that therefore even the simplest phenomenon can be described in many different ways. This basic social constructionist perspective highlights the connection of DA to post-structuralist theories and a rupture with more traditional models of language. The third theme is the concern of DA with the *action orientation* of discourse, whereby discourse analysts see all discourse as social practice, that is, people use it to do things. For example, advertisers sell us products, lifestyles, dreams, and identities. Highlighting this means recognizing that “discourse does not occur in a social vacuum and it is oriented to specific interpretive contexts” [44, p. 59]. Finally, DA treats (multimodal) text as organized rhetorically, in that different discourses compete with each other to establish one version of the world over another.

### 2.2 Musical meanings in multimodal texts

This research examines the degree to which gender-coded elements in sound and music are intentionally used within market segmentation strategies, precisely where music plays a supporting role to other modalities, like in television advertisements. In these instances, the composer’s creative efforts serve a specific purpose, that of accompanying language and moving images. Therefore, a multimodal interpretive framework becomes necessary to explore how narration, video, and music interact.

To approach an understanding of the process of extramusical meaning-making in music, Tagg [39] offers a socio-semantic perspective. In his musical communication model (ibid, p. 174), an “intended message, informed by specifics of transmitter subjectivity in objective relation to the sociocultural field, passes from idea or intention, via its concretion in sonic form (channel) to receivers who respond to what they hear”. In simpler terms, the *transmitter* is any individual producing or reproducing music (e.g. composer, DJ, or performer). *Channel* represents the piece of music or sequence of musical sounds, while *receivers* are those hearing or using the music (might even be the transmitters themselves). The *intended message* is what a transmitter hopes to express. Some examples of verbal approximations of intended (extra)musical messages could be ‘rural loneliness’, ‘noble suffering’, ‘romantic sensuality’, ‘hippy meditation’ or ‘religious wonder’ (ibid, p. 176), which listeners from the same cultural background—sharing a common store of signs and sociocultural norms—should be able to decode. The store of signs is *a part* of the sociocultural norms because it contains “all musical conventions from the relevant culture, as well as all the socially negotiated norms about which elements of music have which connotations and are suited to which purposes”. Tagg avoided presenting the store of signs simply as a subset of the sociocultural norms to better explain the concepts of codal interference and incompetence. Which, in turn, describe two distinct mechanisms that produce major differences between the encoded and decoded messages. *Codal incompetence* arises when transmitter and receiver do not share a common store of signs, while *codal interference* arises when transmitter and receiver differ, even partially, in terms of sociocultural norms or when the music is visually, verbally, socially, or ideologically recontextualized. A highly topical example of how recontextualization gives rise to codal interference is offered by *genderremixer.com*, a web app created by video remix artist Jonathan McIntosh, which allows users to swap the soundtracks of heavily gendered toy commercials (masculine-targeted audio on feminine-targeted video and vice versa). Here, the ensuing humorous effect is precisely due to the codal interference resulting from the visual and ideological recontextualization of the soundtracks. Which, in turn, prompts us to reiterate the importance of the visual and ideological context of music, that is, of its multimodal and discursive nature.

Music and sound are certainly important modes of communication in and of themselves, but they become even more relevant when coupled with other semiotic modes such as image and written language. Especially because music makes up for the limitations of language, “often bringing the most affective aspects to multimodal texts” [46]. Studying music as a multimodal discourse can open up new possibilities for the examination of how different modalities function intersemiotically to mark and maintain identities [47]. According to Bezmer and Jewitt [48], the term ‘multimodality’ has gained popularity in academia since the mid-1990s, spanning across various disciplines including linguistics, semiotics, media studies, and psychology. It acknowledges the diversity and complexity in the ways humans communicate and make meaning. Traditionally focused on speech and writing, linguistics has expanded to include multimodality, aligning with disciplines like psychology and anthropology that study ‘meaning-making’. Various linguistic traditions and methodologies, ranging from discourse analysis to cognitive linguistics, have incorporated the study of multimodality, leading to a broad spectrum of perspectives and approaches in the field. Methodologies vary, with some studies focusing on in-depth single-case analyses and others employing large corpora for hypothesis testing. This diversity reflects the incorporation of elements from different approaches and disciplines. Each discipline, however, has traditionally specialized in studying a specific mode of communication, for example, linguistics focuses on speech and writing, while semiotics is centered more on images and films. Multimodality challenges this specialization, emphasizing the interconnectedness and co-occurrence of different modes in meaning-making. Thus, the study of multimodal discourse focuses on similarities and differences between semiotic modes, as well as how they are incorporated into multimodal texts. A central notion in this context is that of *multimodal genre*, which is used to describe “regular patterns of semiotic choices in multimodal communicative objects and events that are particular to specific communities and cultures” [49]. As discussed in the introduction, commercials aimed at children—thus including their soundtracks—are organized in distinct *gender-based multimodal genres* for their gender-specific target audiences (the above-mentioned specific communities).

### 2.3 Gendered music styles as cognitive schemas

In several empirical studies, sex and gender have been shown to influence the perception of music. Meyers-Levy and Zhu [50] showed that the level of mental resources that one devotes to cognition and gender affect the meanings inferred by consumers from background aesthetic stimuli (in the study both visual and auditory) incorporated in commercials. It has also been shown that gender moderates the influence of loudness on emotional ratings of the music, such that women attributed more positive qualities to music played at a lower volume [51]. Shortly after, Kellaris and Rice [52] demonstrated how musically-induced mood states affected the perception of time significantly more in females than in males. Notably, it has also been shown that women tend to perceive unpleasant music and sound being more fearful, angry, and arousing than men [53].

However, as reported by Gina Rippon [54], studies that seem to prove that sex determines fixed differences in the structure of our brains may have been misinterpreted, overestimated or affected by publication bias. Even the widespread notion that language-processing is bilateral in women and lateralized in men has been debunked [55]. A recent study also showed that gender inequality, rather than sex, is associated with differences in the brains of women and men [56]. In fact, “it isn’t just experiences that can change our brains: attitudes, especially powerful social stereotypes, can too” [54, pp. 30-1]. Just as linguists came to understand ‘women’s language’ [57] and gender as constructed within social discourse, so music theorists might want to move to an understanding of musical meanings as emergent within multimodal discourse, and thus largely depending upon the socialized bodies that produce and hear them, as “our bodies are cultured, we feel music in different ways according to class, gender, ethnicity, race, [ability, Ed.], place and personal experience” [46]. Thus, our sociocultural background not only shapes musical material, but also mediates music processing and perception.

For cognitive psychology, a schema is a *learned* “cognitive structure, a network of associations that organises and guides an individual’s perception” [58]. These cognitive structures include knowledge about ideas or stimuli, as well as those stimuli’s characteristics and the relationships between them. Schemas not only prevent information overload but also “organize one’s perceptual experience into a coherent and intelligible whole” [59, p. 431] (as cited in [60]). More specifically, *gender schemas* [58, 61] guide an individual’s behavior by assimilating or rejecting what is deemed appropriate for their gender. These cognitive structures *evolve* during our life in a recursive fashion, in that they affect memory, perception, and information processing, which in turn, influence the re-creation of subsequent gender schemas. For example, it is more likely for gender schematic individuals to be able to quickly indicate the gender appropriateness of behaviors and activities [62]. As such, schemas can contribute to the formation of—and share many similarities with—*stereotypes* (στερεός [stereos] + τύπος [typos] = solid/firm + impression) but differ from these as schemas are continuously revised. In the following, we introduced the concept of *music-primed gender schemas* by discussing, in parallel, literature in which music genres are theorized as cognitive schemas, and literature that implies the existence of masculine and feminine styles in music.

So far, we have posited that cognitive schemas influence our perception of music, but in a number of studies [63, 64]—see [60] for a more comprehensive list—popular music genres have been themselves theorized as cognitive schemas (from now on *music-primed schemas*) containing extramusical concepts that can be primed when a subject is exposed to the genre’s music. Such schemas form through repeated exposure to the multimodal discourse encompassing music, which is to some extent globalized, but that also varies from culture to culture as a result of glocalization (a lexical blend of globalization and localism) processes [64]. Schema theory has also been used in literary reading and analysis to explain organized “bundles of information and features” such as literary genres (e.g., science fiction, fantasy, horror). A key factor is that these knowledge structures are seen as dynamic. According to Stockwell [65, p. 106-7], “most everyday discourse is schema-preserving, in that it confirms existing schemas. *Where the confirmation is stereotypical, as in much advertising discourse, this is schema reinforcing*”.

While gendered language patterns in text and lyrics may be relatively more straightforward to interpret, gender coding in sound and music ensues from the historical sedimentation, in musical practice, of multimodal associations between gendered meanings in language, visual images, musical structures, or even musical instruments themselves [66]. Some studies have shown that instrumental timbres are consistently associated with masculinity or femininity even when timbres are presented in isolation (e.g. on tape) and not visually linked to a specific musical instrument [67–69]. What is most striking is that the consequences of such stereotyping are consistently observable in ensemble configurations (e.g., orchestras) not only over time but also on a global scale—for a review of gender stereotyping of musical instruments see [70].

In two studies on the gendering of music [71, 72], Sergeant and Himonides hypothesized that musical sounds or the organization of sounds within a composition (from the Western art music tradition) can infer the sex or gender of the performer or the composer. They found significant agreement between raters on the gendering of music, but they did not find any correlation between the gendering of music and the gender of the composers or performers. Therefore, they concluded that gender is not an inherent quality of music, but that masculinity and femininity are comparatively ascribed to music by the listener. Nonetheless, their studies showed that gender schemas equally affect the perception of music in male and female listeners, and that the gendering of music is intersubjective and measurable in terms of tempo, minor/major key, and tonal weight or density (few/many instruments).

Tagg [42] studied the reception of gendered meanings of ten television theme tunes and found high rates of agreement among participants. His study suggests that several concomitant musical dimensions (such as average tempo, rhythmic, and dynamic regularity, presence, or absence of ‘active’ bass lines) may contribute to conveying gendered meanings. In a further refinement of this study [73] Tagg and Clarida collected an extensive set of verbal-visual associations which showed, among other things, that tunes associated with female characters were much more likely to be categorized as quiet and calm.

In a more recent study, Wang and Horvát [74] computationally extracted twelve descriptors of musical parameters and perceptual features (such as tempo, key, time signature, valence, loudness, danceability, acousticness) for more than 200,000 songs by more than 8,000 globally distributed artists, and from across a multitude of popular music genres (571 tags). They found statistically significant differences, concerning the gender of the composers, for eleven out of twelve musical parameters. Thus, their study proved the existence of measurable, supra-genre, gendered music styles in the global music industry.

A clarification is due, in that an apparent incongruence seems to emerge from the literature. On the one hand, we saw how Sergeant and Himonides [71, 72] concluded that, although intersubjective, the gendering of music does not lie in a set of precise musical parameters, and that therefore one cannot infer the gender of a artist/performer from their work. And on the other, Wang and Horvát [74] seemed to have succeeded in that precise task. The reasons for this are manifold: Western art music is much more constricted in its formal styles, and therefore less prone to discursive interpretations than popular music genres; then the sheer number of songs in these studies are not comparable (more than 200,000 against less than 40). Finally, feminine and masculine patterns in the performance and composition of music should be considered on par with gendered registers in spoken language, such as Lakoff’s ‘women’s talk’ [57]. As such, these differences should not be understood in terms of a causal relationship between the gender of the artists and gendered musical patterns. Individuals may tend to use forms of expression that they deem appropriate for their identity, but given the discursive nature of gender we cannot possibly generalize this behavior (i.e., even strong correlation is not causation), as this would end up reinforcing traditional gender discourse and its power relations.

As previously discussed, gender schemas have been proven to mediate our perception of music. But what is even more interesting is that this relationship seems to work somewhat both ways, as music-primed schemas have been empirically shown to alter our perception of other people’s ethnicity, rural/urban background, age, expertise [63] and even gender [75] (as cited in [60]). In a recent study closely related to music-primed schemas, Clausen et al. [76] asked cisgender participants to walk while listening to altered versions of their footsteps that would sound more stereotypically masculine or feminine. Through this subtle auditory-induced embodiment illusions, the authors were able to manipulate the gender identity of participants temporarily.

In summary, scholars from both cognitive poetics [65] and music psychology [63] seem to concur in treating genres as cognitive schemas. Moreover, as previously discussed, the gendering of music ensues from the historical sedimentation of multimodal associations. Thus, we posit that not only traditional gender norms can be understood from the perspective of cognitive psychology in terms of gender schemas [58, 61], but also that masculine and feminine music styles can be interpreted as developing from *music-primed gender schemas*, which themselves are likely formed through repeated exposure to gender-based multimodal genres—see [77, 78] for more details on the relationship between heteronomous/multimodal music listening and the gradual process of enculturation.

Finally, a few words should be spent on how to bridge the proposed interpretive frameworks. Gender is better understood in terms of power relations, and therefore we use the multimodal discourse framework, but the propagation of gender differences in music practice and cognition is better understood in terms of cognitive schemas. The two frameworks are in this sense complementary, and the assumptions on which both are based, do not contradict each other. Understanding music as a multimodal discourse enables us to frame the analysis of the gendered message (*cultural* construction of gender), while gender schemas (*learned* cognitive networks of associations) allow us to explain how gender influences the cognition, perception, and production of the observed gendered musical messages.

## 3 Methods

As already discussed in section 1.2 and Fig 1, we propose an integrative approach [37] combining CA variables, music-focused perceptual responses, and multimodal affective ratings. We organized our data collection in a hierarchical fashion, where at the most factual level we performed content analysis on the commercials. CA is widely used in the industry to assess gender representation [44, p. 43] although critics of this practice have pointed out how CA is unable to distinguish between levels of signification, as it solely highlights how frequently certain categories occur in the analyzed media; thus, it becomes fundamental to complement CA with other analytical tools.

Music is arguably the most affective modality [46] and therefore it makes sense to analyze musical discourses on gender in terms of the emotions that were intentionally encoded in the soundtracks of the commercials. In addition, given that we theorized music as a multimodal discourse, we decided to collect multimodal affective ratings, that is, participants rated each commercial in its entirety (comprising video and sound) on distinct emotion scales. The presence of widespread gender stereotypes on emotions in popular culture lends additional support to this line of inquiry—for a detailed discussion on how emotions function as a gendering construct see [79]. Building upon this idea, analyzing musical discourse in terms of the intended emotions provides a means to explore the gendered musical meanings that are the focus of our analysis.

Finally, the question arises as to how to bridge the most factual level in our dataset (the CA items) with the most subjective one (the multimodal emotions). For this, we proposed to use ‘mid-level perceptual features’ which are used in computational music studies to describe musical characteristics (such as melodiousness, beat strength, rhythmic complexity) that are meant to be musically relevant and instantly identifiable by the majority of listeners. Such features have been shown to have high degrees of consistency across listeners, and they can be predicted to some degree directly from the acoustic signal. In addition, they have been found to correlate with affective dimensions of music [80]—see [81] for a review on music and emotion.

### 3.1 Data collection ethics

The study was approved by the Queen Mary Ethics of Research Committee (Ref. QMERC20.515). All participants were at least 18 years old, resident in the UK, and were recruited between June 21st and July 1st 2022. All participants gave informed consent, whereby the surveys would not load if they did not tick the checkbox in the website to confirm they read the provided consent form and information sheet. Data collection was fully anonymized, in line with the principles expressed in the Declaration of Helsinki.

### 3.2 Sampling method

In March 2022, we initially downloaded 5614 videos from the official YouTube channel of a major UK toy retailer (Smyths Toys Superstores). From these, we then selected only those that had the highest production quality, that is, those that were also most likely intended for television. In this pre-screening process, commercials that did not have any audio, formatted for mobile phones, or which featured a substantial amount of on-screen text were disregarded. This was done to guarantee comparability of our results with previous studies, the vast majority of which were performed on TV commercials. Given that our focus was adverts that were unambiguously gender-targeted, we also dropped all ads featuring toddlers and preschoolers, as these are usually targeted at their parents. In contrast with previous studies where commercials were selected through convenience sampling of TV programming during a limited amount of time [18, 20], we accounted for and substantially minimized duplicates, in that we dropped those videos that had the same title.

**Fig 2:**
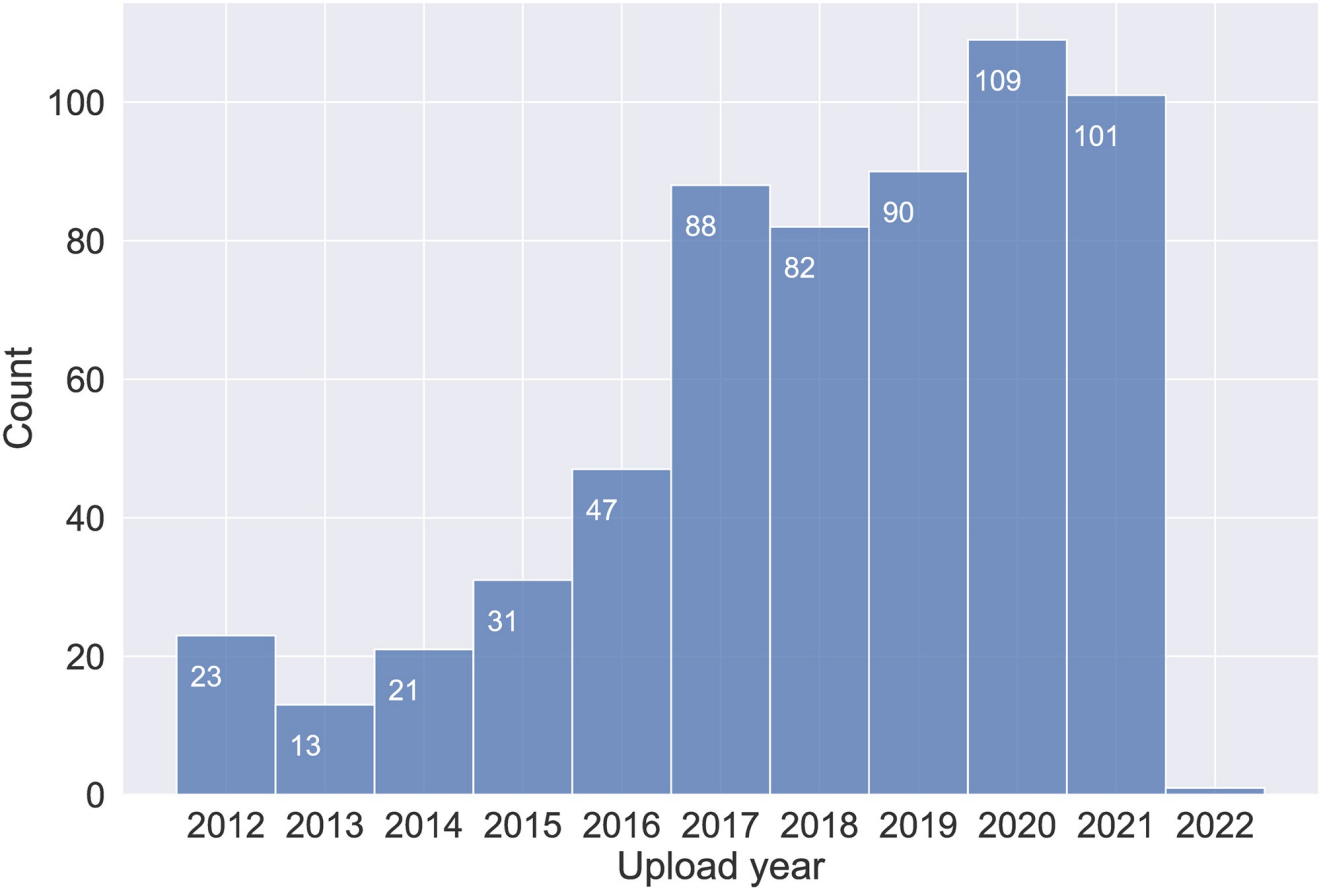
Distribution of the selected commercials per year of upload.

Given that we were not interested in analyzing the content of the retailer’s YouTube channel per se, but we were rather interested in understanding the gendering of sound and music in the toy industry at large, we needed to enforce some balance across gender targets. To achieve this, the remaining commercials were cursorily categorized based on their intended target audience (feminine, masculine, or mixed audience). Such *preliminary* classification was performed following simple heuristics regarding the gender of the majority of presenters featured in the commercial, the color coding of the video and ultimately the category of the product. It comes without saying that such pre-selection process has the potential to introduce bias in the samples, however, it was a necessary step to obtain a balanced dataset for our following analyses. This resulted in 1778 (by and large unique) commercials, 780 of which were tagged as ‘feminine’, 509 as ‘masculine’, and 489 as ‘mixed audience’. Our final sample of 606 commercials was obtained by randomly sampling 200 videos from each of the above categories, plus 6 additional control stimuli used for computing the inter-rater agreement scores of the perceptual ratings. The upload dates span 10 years, from 2012 to 2022 (Fig 2), which, to the best of our knowledge, constitutes the longest time frame in the literature for this kind of study.

### 3.3 Content analysis

In this study, the *gender orientation* (also *target* audience) of the commercials was determined by the gender of the actors/presenters. Following Johnson and Young [19], to account for tokenism, whenever a presenter of the other gender was included in the background or for just a few seconds, these were considered token gender representations and not explicit market orientations. All fictional characters, even when realistic (e.g. from a video game), were not considered actors/presenters, and the corresponding commercials were coded as having no actors. Whenever commercials featured hands without a face or head, these were also coded as having no actors.

In addition, four distinct items describing the sound of the voices in the commercial were collected using a coding schema based on Verna’s research [14]. But unlike the original work, we coded for all voices in the commercial, both diegetic and non-diegetic. Commercials were coded in terms of the *type of voices* (“Spoken”, “Sung”, “Both spoken and sung”, “No voices”), then in terms of *voices age* (“Adults” which included young adults, “Children”, “Children and adults”, “No voices”), *gender exaggeration* of the voices [19] (“All normal sounding”, “Exaggeratedly masc.”, “Exaggeratedly fem.”, “No voices”), and finally in terms of *voice gender* (“Feminine”, “Masculine”, “Feminine and masculine”, “Unclear”, “No voices”), but as there was only a single case in which the gender of the voices was unclear, we merged this in “Feminine and masculine.”

To determine the reliability of each variable, 15% of the commercials were double-coded by two coders (M, 33; W, 28) independently, as done in the research by Kahlenberg and Hein [20]. A Krippendorff’s alpha level of.91 was reached for the gender orientation of the commercials, .81 for voice type, .87 for voice age, .96 for voice gender, .81 for voice gender exaggeration; and therefore, met the standards of reliability required for this type of analysis [37].

### 3.4 Music-focused mid-level perceptual features

Unfortunately, most of the existing platforms for large-scale data annotation, such as Amazon MTurk, are well-known places of exploitation [82, 83]. Therefore, we decided to use Prolific.co to recruit participants, as they endorsed a principle of ethical reward. Participants in our study were paid between 7 and £8 per hour. To minimize the effects of careless responding, a low-effort metric was computed by summing the length of all long strings for each participant, and those that scored above two standard deviations from the average value were screened out during data collection, as it was performed in batches of 50 participants.

Musically trained participants–-i.e., with at least three years of experience with an instrument—rated the soundtracks of the commercials (i.e., sound only) on 15 music-focused bipolar scales based on perceptual studies of music [84, 85]. Each participant rated 30 commercials that were randomly and dynamically selected based on the number of ratings previously collected for each commercial in the dataset. More formally, each stimulus was randomly sampled from the subset of commercials that at the moment of retrieval had less than 6 ratings. Given that the same stimulus could be presented to different participants during data collection, this resulted in a slightly uneven distribution of the ratings. For the 606 commercials and for each of the scales, we collected a total of 4560 ratings from 152 participants from the UK (75 M, 77 F, aged 40 ± 14). Of these ratings, 912 were for 6 control stimuli that were rated by all participants. Six ratings were collected instead for 560 of the remaining 600 commercials, five ratings for only six of them, and more than six ratings (with a maximum of twelve) were collected for the rest.

Given that our focus was on music, but soundtracks in commercials consisted of speech, music and sound effects, our question was formulated as follows: “The following is a series of perceptual attributes of music. You are asked to evaluate the *music in the background* in terms of the adjectives on each side of the scale”. To estimate the degree of consistency of each scale we computed the interrater agreement score $r_{wg}^{*}$ [86] on each of the control stimuli separately. The collected scales were Electric/Acoustic with an average $r_{wg}^{*}$ of.74 across the control stimuli, Distorted/Clear (.78), Many/Few instruments (.79), Loud/Soft (.77), Heavy/Light (.85), High/Low pitch (.83), Wide/Narrow pitch variation (.79), Punchy/Smooth (.80), Harmonious/Disharmonious (.82), Clear melody/No melody (.81), Repetitive/Non-repetitive (.79), Complex/Simple rhythm (.79), Fast/Slow tempo (.88), Dense/Sparse (.83), and Strong/Weak beat (.79).

### 3.5 Multimodal aesthetic emotions

According to Cowen et al. [87], categorical and dimensional models of emotions that have been extensively used in studies of music emotion do not allow to adequately distinguish between different emotions. For this study, we drew from the aesthetic emotions scale (AESTHEMOS, [88]), which was devised from an extensive review of emotion measures from different domains such as music, literature, film, painting, advertisements, design, and architecture, and thus, was ideal in its flexibility for our use with multimodal stimuli.

Given that our focus was on music and sound, in a previous study we limited our choice to a subset of 10 AESTHEMOS subscales that intersected with the 13 emotions listed by Cowen et al. [87], namely ‘Happy or delightful’ (average $r_{wg}^{*}$ of.74), ‘Amusing or funny’ (.72), ‘Ugly or distasteful’, ‘Beauty or liking’ (.73), ‘Calm or relaxing’ (.79), ‘Worrying or oppressive’, ‘Energizing or invigorating’ (.77), ‘Angry or aggressive’ (.84), ‘Sad or depressing’, and ‘Triumphant or awe-inspiring’ (.71). We used a single unipolar item for each subscale, instead of two. Of these 10 scales, 7 showed significant discriminant capabilities, and therefore, we dropped the three negative emotion scales that in the pilot were proven to be irrelevant, namely ‘Ugly or distasteful’, ‘Worrying or oppressive’ and ‘Sad or depressing’. For the 606 commercials and for each of the 7 emotion scales, we collected a total of 4530 ratings from 151 participants from the UK (76 M, 75 F, aged 39 ± 13) following the same procedure implemented for the music-focused descriptors. Of these ratings, 906 were for the previously mentioned control stimuli, on which we computed the interrater agreement score $r_{wg}^{*}$ [86] reported above for each scale in parenthesis. Six ratings were collected for 569 of the remaining 600 ads, five ratings for twelve of them, and more than six ratings (with a maximum of fourteen) were collected for the rest. Given that we aimed to analyze the intended emotional profile, our question was formulated as follows: “Toys commercials are targeted at an audience mainly consisting of children and aim at evoking the following emotions. Pay attention to both sound and images and rate each *intended* emotion accordingly (1 for ‘not at all’, 7 for ‘very’)”.

For this pool of participants, we also collected additional information on their gender identity, sexual orientation, gender-role self-concept [89], subjective socioeconomic status [90], ethnicity, and disability. This has helped us to detect underrepresented sub-populations in our data, as later discussed. This data, although not within the scope of the current study, can help in future research to investigate gender intersectionality in the perception of aesthetic stimuli [91].

### 3.6 Data analysis

As shown in Fig 1, numerous methods of data analysis and testing were employed in this study: Pearson’s chi-square tests were performed to verify the dependency of voice-related CA variables on the gender target of the commercials; ANOVA was performed to estimate the significance of the influence of gender-based market segmentation on music-perceptual and affective scales; UMAPs were computed to obtain intelligible two-dimensional (interactive) visualizations of the music-perceptual and affective scales, especially in relation to the gender target of the commercials; EFA was used to reveal the latent unmeasured constructs (factors) that group together the music perceptual scales; the former in turn served as a dimensionality reduction step for the regression analysis, which was performed on the multimodal aesthetic emotions to understand the influence of the auditory channel represented by the estimated factor scores and the voice-related CA (dummy) variables. In all of the related analyses, to obtain one value per stimulus for each of the music-perceptual and affective scales, we aggregated each commercial’s collected ratings by computing their mean.

#### Contingency tables and chi-square test

Pearson’s *χ*^2^ test of independence checks if two variables are independent of each other by examining the collected data, such as between the voice-related CA variables and the gender orientation displayed in Table 1. A low p-value (a high *χ*^2^ test value) corresponds to the rejection of the null hypothesis, which assumes the independence of the two variables.

**Table 1:** Contingency tables of voice-related CA variables with *χ*^2^ tests of independence. The column “All” includes commercials without actors or presenter (N = 94).

All N = 606		Feminine N = 163	Masculine N = 149	Mixed N = 200
	**Type**	*χ*^2^(6, *N* = 512) = 89.02, *p* = .000		
5.6%	Sung	9.8%	None	2.5%
18.8%	Spoken and sung	36.8%	6.0%	17.0%
67.7%	Spoken	52.8%	81.2%	75.5%
7.9%	No voices	0.6%	12.8%	5.0%
	**Age**	*χ*^2^(6, *N* = 512) = 39.51, *p* = .000		
79.5%	Adults	76.7%	79.2%	83.0%
5.9%	Children and adults	8.0%	6.0%	6.5%
6.6%	Children	14.7%	2.0%	5.5%
7.9%	No voices	0.6%	12.8%	5.0%
	**Gender**	*χ*^2^(6, *N* = 512) = 332.1, *p* = .000		
39.8%	Feminine	95.7%	2.0%	29.5%
46.9%	Masculine	1.8%	83.9%	54.5%
5.4%	Feminine and masculine	1.8%	1.2%	11.0%
7.9%	No voices	0.6%	12.8%	5.0%
	**Gender exaggeration**	*χ*^2^(6, *N* = 512) = 243.6, *p* = .000		
16.5%	Exagg. feminine	44.8%	None	8.0%
15.5%	Exagg. masculine	None	40.3%	7.0%
60.1%	All normal sounding	54.6%	47.0%	80.0%
7.9%	No voices	0.6%	12.8%	5.0%

#### ANOVA

A one-way analyses of variance (ANOVA) assesses whether there are statistically significant differences in the means across two or more groups. The null hypothesis assumes that all group means are identical. If the p-value is sufficiently low, it indicates that at least one group mean is different from the others. We performed between-targets (i.e., gender targets of the commercials) one-way ANOVAs for each of the collected scales reported in Tables 2 and 3. When ANOVA (F-test) assumptions were violated, we performed a Kruskal-Wallis H-test instead.

**Table 2:** Between gender orientations, one-way Kruskal-Wallis (H-test) or ANOVA (F-test) of the music-focused scales. The three rightmost columns show the average values within each group of commercials.

Perceptual scale	Statistic	p	Fem	Masc	Mix
**Electric/Acoustic**	H = 42.0	.000	3.4	2.7	3.4
**Distorted/Clear**	H = 137.1	.000	5.2	3.6	4.6
**Many/Few Instruments**	H = 22.3	.000	4.6	4.1	4.3
**Loud/Soft**	H = 83.5	.000	4.2	3.0	3.9
**Heavy/Light**	H = 203.5	.000	5.2	3.0	4.6
**High/Low pitch**	H = 161.8	.000	3.3	4.6	3.8
**Wide/Narrow pitch var**.	H = 1.3	.533	4.4	4.4	4.3
**Punchy/Smooth**	H = 80.3	.000	3.8	2.8	3.4
**Harm./Disharmonious**	H = 79.9	.000	3.0	3.9	3.5
**Clear melody/No melody**	H = 51.3	.000	3.0	3.8	3.6
**Repetitive/Non-repetitive**	H = 3.4	.184	2.9	3.1	3.0
**Complex/Simple rhythm**	F = 22.2	.000	5.0	4.4	4.7
**Fast/Slow tempo**	F = 2.1	.122	3.4	3.2	3.3
**Dense/Sparse**	H = 107.9	.000	4.2	3.2	3.9
**Strong beat/Weak beat**	H = 19.1	.000	3.6	3.1	3.5

**Table 3:** Between gender orientations, one-way Kruskal-Wallis (H-test) or ANOVA (F-test) of the emotion scales. The three rightmost columns show the average values within each group of commercials.

Emotion	Statistic	p	Fem	Masc	Mix
**Happy or delightful**	H = 124.0	.000	5.1	3.9	5.0
**Amusing or funny**	H = 73.7	.000	3.2	3.0	3.9
**Beauty or liking**	H = 242.8	.000	4.2	2.4	3.2
**Calm or relaxing**	H = 143.8	.000	3.2	1.9	2.7
**Energizing or invigorating**	H = 139.2	.000	3.5	4.8	4.3
**Angry or aggressive**	H = 185.2	.000	1.2	2.6	1.5
**Triumphant or awe-inspiring**	H = 102.5	.000	2.6	3.5	3.2

#### UMAP

**Fig 3:**
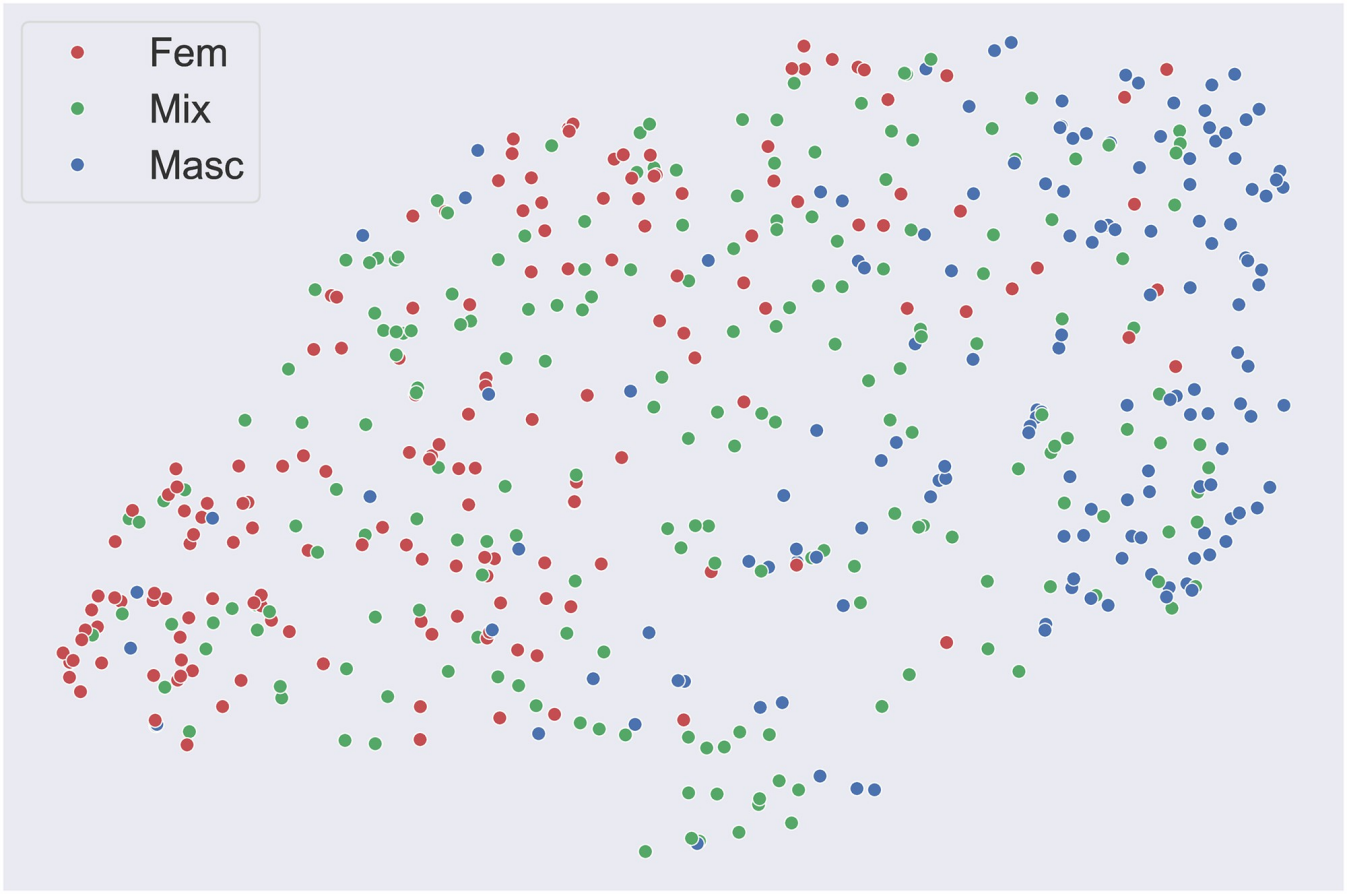
Non-linear dimensionality reduction (via UMAP) of the music-focused scales. A clickable interactive version with the original YouTube links is provided at marinelliluca.github.io/mf-interactive.

**Fig 4:**
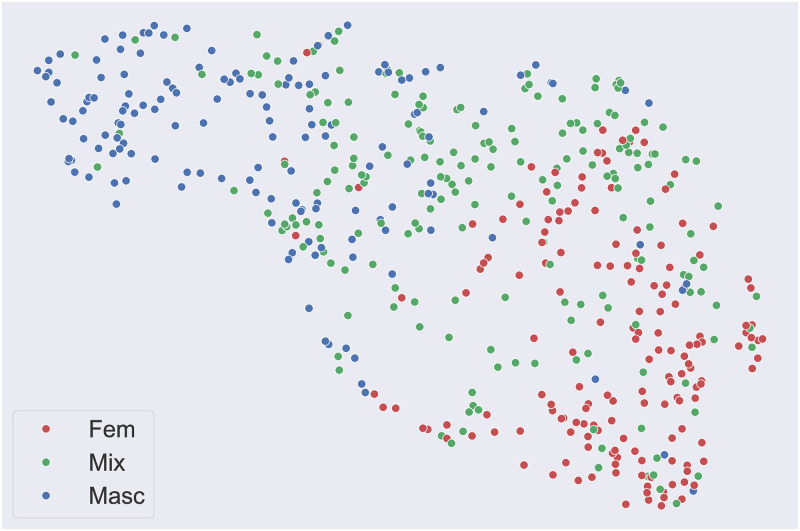
Non-linear dimensionality reduction (via UMAP) of the emotion scales. A clickable interactive version with the original YouTube links is provided at marinelliluca.github.io/mm-interactive.

As shown in Figs 3 and 4, non-linear dimensionality reduction was computed via uniform manifold approximation and projection (UMAP, [92]) to better appreciate the degree of gender polarization introduced by market segmentation in the soundtracks of the commercials. In simple terms, starting from a high dimensional graph representation of the data, UMAP allows to compute a lower-dimensional representation to be as structurally similar as possible to the initial graph. The resulting dimensions are complex non-linear transformations of the initial numerical descriptors—which in our case are the music-focused scales or the aesthetic emotions—and therefore are not easily interpretable in and of themselves. What is important, though, is that UMAP allows the exploration of high-dimensional data, as clusters and other patterns become intelligible and visible once transposed onto a two or three-dimensional space.

#### Exploratory factor analysis

As previously argued, adverts for feminine, masculine and mixed audiences were organized in multimodal genres, and we hypothesized that these genres would produce substantially different underlying latent structures in the music-focused scales. Therefore, using the R “psych” package, we conducted a distinct exploratory factor analysis (EFA) on each of the target audiences separately. To ensure the appropriateness of the data for EFA we first performed Bartlett’s test of sphericity and the Kaiser–Meyer–Olkin test. Due to high multi-collinearity, which was detected by calculating the variance inflation factor (VIF), we had to exclude the scale Heavy/Light from EFA and the following regression analysis, as this scale had a VIF of 7.1, while all remaining variables displayed VIF values below 5. We then conducted a modified parallel analysis with Monte Carlo estimates using the “paran” package, as suggested by Glorfeld [93], and compared the Bayesian information criterion (BIC) scores of each model for different numbers of factors, to avoid over-retention (BIC promotes parsimony). Given that, as expected, the data did not pass the normality test (Mardia’s tests for skewness and kurtosis), we opted for an iterated principal axis factor extraction [94], and used Spearman’s correlation matrices as input for both parallel analysis and EFA [95]. In addition, as factors were assumed to be correlated with each other, we opted for an oblimin rotation. Pattern coefficients were considered to be salient when ≥ 0.5.

#### Regression analysis

As shown in different analyses within this manuscript, the multimodal affective scales resulted in being extremely gender-polarized. Such polarization proved to be intractable, as conducting a regression analysis across the three target audiences would have violated several assumptions of ordinary least squares (OLS). Some of the scales were so skewed that the central normality of the residuals was not met even after transforming the dependent variable to central normality (which was done following [96]). Therefore, we decided to fit different linear models for each target audience (with the “lm” function). Each emotion was modeled as a linear combination of four voice-related categorical variables (type, age, gender, and gender exaggeration of the voices) and the extracted music-focused factors (three or four, depending on the target audience). Finally, in order to compute a realistic estimate of the proportion of variance explained by each regressor, we used the “drop1” function to evaluate the marginal sum of squares (SS) for each regressor by comparing the full model with a model without the regressor under analysis (Type III SS).

## 4 Findings

### 4.1 Content analysis

In terms of the gender orientation of the 606 analyzed commercials, 163 were coded as targeted at a feminine audience, 149 at a masculine, 200 at a mixed audience, and the remaining 94 featured no actors or presenters. Given that we performed a preliminary selection to ensure balance across gender targets, these results will be used as ground truth in following analyses, rather than to determine the degree of (un)balance within the chosen media channel (cf. [20]). For each of the following contingency tables, a chi-square test of independence was performed.

As reported in Table 1, spoken voices dominated (67.7%), followed by singing voices (24.4%), and a small percentage had no voices (7.9%). Commercials aimed at a feminine audience prominently featured singing voices (46.6%) and spoken voices (52.8%), with one instance of no voice (0.6%). Masculine-targeted commercials predominantly used spoken voices (81.2%), with a much smaller presence of singing voices (6%) and some without voices (12.8%). Mixed audience commercials predominantly featured spoken voices (75.5%), with fewer singing voices (19.5%) and a small fraction without voices (5%).

The majority of commercials employed adult voices (79.5%), with children’s voices appearing alone or with adults in a small fraction of ads (5.9% and 6.6%, respectively). Feminine-targeted commercials featured a higher proportion of children-only voices (14.7%) compared to masculine (2%) and mixed audience commercials (5.5%).

Masculine-only voices were most common (46.9%), followed by feminine-only voices (39.8%), and a combination of feminine and masculine voices (5.4%). Gender-targeted commercials predominantly featured voices of the target gender, while mixed audience commercials mainly featured masculine-only voices (54.5%), with fewer instances of feminine-only (29.5%) and combined gender voices (11%).

Gender exaggeration was fairly common, with similar rates for feminine (44.8%) and masculine (40.3%) voices in their respective market segments. However, gender exaggeration was significantly less frequent in commercials for mixed audiences, at 8% for feminine voices and 7% for masculine voices.

### 4.2 Music-focused perceptual ratings

Significant polarization emerged for twelve out of fifteen scales, where stark contrasts can be observed in Table 2 between feminine and masculine-targeted commercials, with commercials targeted at mixed audiences generally registering in-between values. Masculine adverts were more “Electric” than “Acoustic”, more distorted, disharmonious and with a less clear melodic contour than feminine ones. They also were denser in terms of instrumentation (also see the “Many/Few instruments” scale), “Punchier”, with stronger beats, and, therefore, were generally louder and perceived as heavier. Also in terms of rhythmic complexity, they were more complex than the feminine-targeted commercials. A clear picture then emerged, in which the soundtracks in boys’ adverts were significantly more *abrasive* than those in girls’ ads.

As shown via UMAP in Fig 3, feminine (in red, on the left) and masculine-targeted commercials (in blue, on the right) appear to form two very distinct clusters. Mixed audience commercials, instead, seem to be more uniformly distributed in the space of the music-focused scales.

### 4.3 Multimodal aesthetic emotions

On the emotion scales, we performed the same set of analyses that we applied on the music-focused ratings. As shown in Table 3, significant polarization emerged for all the collected scales, which came as no surprise as we previously ran a pilot study to specifically avoid collecting redundant or irrelevant high-level data. Similar to the music-focused scales, stark contrasts can be observed between feminine and masculine-targeted commercials, with commercials targeted at mixed audiences often registering in-between values. Commercials targeted at the masculine audience were the least “Happy or delightful”, the least “Amusing or funny”, “Calm or relaxing”, and registered the lowest values on the scale “Beauty or liking”. They instead were the most “Energizing or invigorating”, “Angry or aggressive”, and “Triumphant or awe-inspiring”. Apart from the scale “Amusing or funny”, which scored the highest values within mixed audience commercials, adverts targeted at the feminine audience displayed an opposite behavior to the masculine ones. For example, they were the most “Calm or relaxing” and the least “Angry or aggressive”. As previously highlighted with the music-focused scales, masculine-targeted commercials appeared again to be significantly more abrasive than the feminine ones. We then performed non-linear dimensionality reduction of the 7 emotion scales with UMAP. In Fig 4, feminine (in red, on the right) and masculine-targeted commercials (in blue, on the left) appeared again to form two very distinct clusters, with mixed audience commercials more uniformly distributed in the emotion space.

### 4.4 Factor scores regression analysis

In our final set of analyses, we explored how each emotion scale can be explained in terms of auditory-related variables from both the CA and the music-focused sets. In addition, we first performed an EFA on the music-focused scales for improved interpretability, as discussed in section 3.6, and then used the obtained factors scores to perform the regression analysis via OLS.

#### Exploratory factor analysis

As shown in Table 4, the music-focused scales followed three latent dimensions (PA1, PA2, PA3, arranged in descending order of magnitude) that turned out to be consistent across all target audiences. Within masculine-targeted ads, however, the soundscape appeared to be influenced by an additional fourth axis (PA4), thereby confirming our hypothesis on the influence of *gender-based multimodal genres*. The first factor was positively saliently loaded, or nearly so, by the same scales across all targets. Namely, “Electric/Acoustic”, “Loud/Soft”, “Punchy/ Smooth”, “Fast/Slow tempo”, “Dense/Sparse”, and “Strong beat/Weak beat”. We could then rename PA1 as the “Energizing/Soothing” axis. The second factor instead was interpretable in terms of harmoniousness and melodiousness, although it also entailed an element of abrasiveness as well, as for both masculine and mixed audiences it was also saliently loaded by the scale “Distorted/Clear”. On the other hand, PA3 seems to be more difficult to interpret, as it was saliently loaded by “Many/Few instruments” and “Wide/Narrow pitch variation” across all targets, and these two scales did not easily fit conceptually with each other. But when we looked at “Repetitive/Non-repetitive” and “Complex/Simple rhythm”, we noticed that these scales were as well saliently loaded on that factor across all targets (or nearly so). Therefore, PA3 was associated with the complexity of rhythm, instrumentation, and melody. Finally, PA4 was strongly attending to pitch height. Interestingly, we could see how the scale “High/Low pitch” scores extremely high in uniqueness (*u*^2^, the ratio of variance not explained by the factors) within feminine and mixed audience commercials, whereas within masculine-targeted adverts it was relevant enough to almost constitute a factor on its own.

**Table 4:** Pattern coefficients and uniqueness (*u*^2^) for the principal axis (PA) factors computed on music-focused ratings of feminine-, masculine-targeted, and mixed audience commercials. Salient loadings in bold (when ≥ .5).

	Feminine-targeted ads				Masculine-targeted ads					Mixed audience ads			
	PA1	PA2	PA3	*u* ^2^	PA1	PA2	PA3	PA4	*u* ^2^	PA1	PA2	PA3	*u* ^2^
**Electric/Acoustic**	**.66**	-.24	-.02	.45	**.63**	-.42	-.25	.11	.46	.43	**-.53**	-.25	.47
**Distorted/Clear**	.47	-.43	.09	.52	.45	**-.67**	.04	-.04	.35	.28	**-.77**	.11	.33
**Many/Few Instruments**	.20	.19	**.62**	.49	.20	.20	**.60**	-.01	.54	.39	.08	**.52**	.59
**Loud/Soft**	**.79**	.04	.13	.32	**.76**	-.11	.21	-.09	.32	**.83**	-.11	.17	.29
**High/Low pitch**	-.31	.17	.05	.87	-.11	.08	.04	**.83**	.24	-.13	.49	.08	.74
**Wide/Narrow pitch var.**	-.05	.14	**.65**	.56	.17	.09	**.66**	.42	.34	.13	.26	**.69**	.46
**Punchy/Smooth**	**.90**	-.10	.03	.15	**.84**	-.04	.04	.00	.29	**.77**	-.12	-.01	.39
**Harm./Disharmonious**	-.22	**.77**	.08	.32	-.02	**.82**	.05	.07	.29	.03	**.86**	.02	.27
**Clear melody/No melody**	.13	**.94**	.02	.13	.22	**.82**	-.02	.09	.22	.25	**.80**	-.12	.27
**Repetitive/Non-repetitive**	**.62**	.13	-.47	.52	.43	.24	**-.64**	.11	.36	.43	.30	**-.54**	.40
**Complex/Simple rhythm**	.29	-.19	.47	.59	.15	.03	.49	-.01	.73	.24	-.08	**.51**	.68
**Fast/Slow tempo**	**.71**	-.04	.02	.48	**.54**	.12	-.11	.02	.69	**.52**	.15	.02	.71
**Dense/Sparse**	**.60**	-.04	.41	.38	**.61**	.08	.41	-.27	.30	**.68**	-.13	.33	.43
**Strong beat/Weak beat**	**.91**	.16	-.04	.20	**.77**	.30	-.12	-.13	.26	**.81**	.22	-.17	.25

Factor score indeterminacy is a well-known issue in factor analysis, where an infinite set of factor score estimates can be computed, which would lead to the same solution (i.e., factor loadings). This problem has been known for almost 100 years and has yet to be solved [97]. To minimize bias, we computed the music-focused factors scores for each of the commercials using the *ten Berge* estimation method, as previous studies showed that the scores obtained with this approach best captured the true between-factor correlation as well as the correlation of an extracted factor with a external variable [98].

#### Regression analysis

In Tables 5–7, we reported only the results of the regression models that passed the F-test of overall significance (*α* = .05), where each table is specific to a certain target audience. We did not report the value of the coefficients as we were only interested in their sign, whereas significant intercepts of the categorical variables were reported only in text. In a few cases, models with interactions between regressors were slightly, but significantly better than the simple model. Nonetheless, these interactions were inconsistent across the target audiences and therefore uninformative, so we did not report them.

**Table 5:** Significant results of ordinary least squares regression performed on the emotion scales (responses in the leftmost column) within feminine-targeted commercials. p < .1, * p < .05, ** p < .01, *** p < .001.

**Amusing**	*R*^2^ = .127, Adj. *R*^2^ = .081, F = 2.8 on 8 and 153 DF, p = .007			
	**Regressor**	**Sign of coeff.**	**Expl. var.**	**F / p**
	**PA1**	-	6.6%	11.5 / ***
**Calm**	The dependent variable was transformed to central normality *R*^2^ = .383, Adj. *R*^2^ = .351, F = 11.9 on 8 and 153 DF, p < .001			
	**Regressor**	**Sign of coeff.**	**Expl. var.**	**F / p**
	**Gender exagg.**	NA	3.2%	7.9 / **
	**PA1**	+	19.4%	48.2 / ***
**Energizing**	*R*^2^ = .273, Adj. *R*^2^ = .235, F = 7.2 on 8 and 153 DF, p < .001			
	**Regressor**	**Sign of coeff.**	**Expl. var.**	**F / p**
	**Voices type**	NA	2.2%	4.5 / *
	**Voices age**	NA	2.3%	4.8 / *
	**Gender exagg.**	NA	1.4%	3.0 /.
	**PA1**	-	11.2%	23.7 / ***
	**PA3**	-	3.1%	6.4 / *
**Triumphant**	*R*^2^ = .099, Adj. *R*^2^ = .052, F = 2.1 on 8 and 153 DF, p = .039			
	**Regressor**	**Sign of coeff.**	**Expl. var.**	**F / p**
	**Voices type**	NA	2.8%	4.8 / *
	**Voices age**	NA	1.8%	3.1 /.
	**Voices gender**	NA	3.0%	2.5 /.
	**PA3**	-	2.9%	5.0 / *

**Table 6:** Significant results of ordinary least squares regression performed on the emotion scales (responses in the leftmost column) within masculine-targeted commercials. p < .1, * p < .05, ** p < .01, *** p < .001.

**Happy**	*R*^2^ = .267, Adj. *R*^2^ = .214, F = 5.0 on 10 and 138 DF, p < .001			
	**Regressor**	**Sign of coeff.**	**Expl. var.**	**F / p**
	**Voices type**	NA	3.6%	6.7 / *
	**Gender exagg.**	NA	3.5%	6.6 / *
	**PA2**	-	2.6%	4.8 / *
	**PA3**	+	1.8%	3.4 /.
	**PA4**	-	1.5%	2.8 /.
**Beauty**	*R*^2^ = .21, Adj. *R*^2^ = .153, F = 3.7 on 10 and 138 DF, p < .001			
	**Regressor**	**Sign of coeff.**	**Expl. var.**	**F / p**
	**Voices type**	NA	4.8%	8.3 / **
	**Voices gender**	NA	4.6%	4.1 / *
	**PA1**	+	2.7%	4.7 / *
	**PA2**	-	3.5%	6.1 / *
	**PA3**	+	1.8%	3.1 /.
**Energizing**	*R*^2^ = .178, Adj. *R*^2^ = .118, F = 3.0 on 10 and 138 DF, p = .002			
	**Regressor**	**Sign of coeff.**	**Expl. var.**	**F / p**
	**PA1**	-	5.5%	9.3 / **
	**PA2**	+	4.5%	7.5 / **
**Angry**	The dependent variable was transformed to central normality *R*^2^ = .384, Adj. *R*^2^ = .339, F = 8.6 on 10 and 138 DF, p < .001			
	**Regressor**	**Sign of coeff.**	**Expl. var.**	**F / p**
	**Gender exagg.**	NA	13.8%	31.0 / ***
	**PA1**	-	6.2%	13.9 / ***
	**PA2**	+	3.6%	8.1 / **

**Table 7:** Significant results of ordinary least squares regression performed on the emotion scales (responses in the leftmost column) within mixed audience commercials. p < .1, * p < .05, ** p < .01, *** p < .001.

**Happy**	*R*^2^ = .206, Adj. *R*^2^ = .164, F = 4.9 on 10 and 189 DF, p <0.001			
	**Regressor**	**Sign of coeff.**	**Expl. var.**	**F / p**
	**Voices type**	NA	2.3%	5.6 / *
	**Gender exagg.**	NA	4.3%	5.1 / **
	**PA2**	-	1.6%	3.7 /.
**Beauty**	*R*^2^ = .244, Adj. *R*^2^ = .204, F = 6.1 on 10 and 189 DF, p <0.001			
	**Regressor**	**Sign of coeff.**	**Expl. var.**	**F / p**
	**Voices type**	NA	2.1%	5.2 / *
	**Gender exagg.**	NA	3.7%	4.6 / *
	**PA1**	+	1.2%	3.0 /.
	**PA2**	-	3.1%	7.8 / **
**Calm**	*R*^2^ = .258, Adj. *R*^2^ = .218, F = 6.6 on 10 and 189 DF, p <0.001			
	**Regressor**	**Sign of coeff.**	**Expl. var.**	**F / p**
	**Voices gender**	NA	2.6%	3.3 / *
	**PA1**	+	6.8%	17.3 / ***
	**PA2**	-	4.9%	12.6 / ***
**Energizing**	*R*^2^ = .200, Adj. *R*^2^ = .158, F-stat. = 4.7 on 10 and 189 DF, p <0.001			
	**Regressor**	**Sign of coeff.**	**Expl. var.**	**F / p**
	**PA1**	-	6.7%	15.9 / ***
	**PA2**	+	1.5%	3.5 /.

Being the most abstract aesthetic emotions in our set, models fitted on the “Amusing” and “Triumphant” scales obtained the worst fits, with adjusted *R*^2^ values well below 0.10 in both cases. Models on the “Beauty” scale, instead, performed surprisingly better, with the highest adjusted *R*^2^ of 0.20. Closely above, we find the scales “Happy” and “Energizing”, with adjusted *R*^2^ values of 0.21 and 0.24 respectively. Whereas the best performing models were fitted on the “Angry” and “Calm” scales, with adjusted *R*^2^ values of 0.34 and 0.35 respectively.

The “Happy” scale appeared to have three common (marginally) significant regressors across masculine and mixed audience commercials, namely the type of voices in the commercials (happier when singing voices were present), the gender exaggeration of the voices (less happy when masculine voices were gender exaggerated), and the extracted factor PA2, which, in both cases, had a negative coefficient and therefore indicated that the less harmonious and melodic the soundtrack, the less happy an advert was perceived. Within masculine-targeted commercials, also PA3 and PA4 appeared to be marginally significant regressors, indicating that simpler and higher-pitched soundtracks led to happier commercials. For the “Amusing” scale we could only report results within feminine-targeted commercials, with PA1 explaining almost 7% of the variance, and its negative coefficient indicating that the more energizing the soundtracks the more amusing the commercials. The “Beauty” scale, instead, appeared to have three common regressors across masculine and mixed audience commercials, namely the type of voices, PA1 and PA2, where soundtracks that were more soothing, melodious and harmonic, with singing voices leading to commercials that scored higher on this scale. Within masculine commercials, the presence of feminine voices and simpler soundtracks also correlated with higher perceived beauty. For the “Calm” scale within feminine-targeted commercials, a positively-signed PA1 explained 19% of the variance (7% within mixed audiences), indicating the validity of the extracted factor and the importance of the soundtracks concerning the level of arousal. The presence of feminine-exaggerated voices in feminine-targeted commercials was associated with lower values on the scale. Within mixed audience commercials, a negatively-signed PA2 also indicated that the more melodious and harmonic a soundtrack, the calmer the ads, and the presence of both masculine and feminine voices was associated with the least calm commercials. For “Energizing”, PA1 was the sole common regressor across all target audiences. Within feminine-targeted commercials, the presence of singing voices and children’s voices had a negative intercept, and feminine-exaggerated voices a positive one. Whereas, within masculine and mixed audience adverts, PA2 had a positive coefficient, indicating that less harmonious and more distorted soundtracks result in more energizing ads. Residuals of the models fitted on the “Angry” scale within feminine and mixed audiences were not normally distributed even after transforming the dependent variable to central normality. Moreover, within masculine-targeted adverts, the presence or absence of gender-exaggerated voices alone explained almost 14% of the variance. Also, a negatively-signed PA1 and a positively-signed PA2 together explained another 10%, where energizing and disharmonious soundtracks with masculine-exaggerated voices led to the most aggressive commercials. For the “Triumphant” scale, we only obtained an extremely low *R*^2^ within feminine-targeted commercials. Singing and children’s voices had a negative intercept, the presence of masculine voices a positive one, and because of a negatively-signed PA3, more complex soundtracks led to more triumphant adverts.

## 5 Discussion

This study investigated the extent to which gender coding may be deliberately used when music is secondary to other modalities and serves a clear purpose, such as in advertisement. By inferring the gender target of toy adverts via content analysis, we gained a more objective standpoint from which to analyse the gendering of music. For this goal, we proposed an integrative approach combining content analysis, multimodal aesthetic emotion ratings, and music-focused perceptual ratings. Musically experienced participants rated the soundtracks of 606 toy commercials on 15 music-focused perceptual scales. A distinct pool of participants rated each commercial (video and audio) on seven emotion scales. Extreme polarization—concerning the gender orientation of the commercials—was found in both multimodal aesthetic emotion ratings and in ratings of music perceptual features. In agreement with previous studies, music in masculine-targeted commercials was found to be significantly more abrasive than that in feminine adverts. The tendency in masculine-targeted commercials to feature more noise, louder music, and more sound effects than feminine-targeted or mixed audience adverts was already documented by Welch et al. [15]. Little seems to have changed more than 40 years later, as commercials targeted at the masculine audience were the loudest, most distorted, most disharmonious, and least melodious in our sample. Rather than being ascribed to music solely by listeners as suggested in [72]—a conclusion that was essentially based on a fallacy as discussed in Section 2.3, masculine and feminine music styles in toy commercials appeared to emerge as a result of deliberate marketing strategies—being ultimately tied to the gender target of the commercials—and reflect traditional gender stereotypes still dominant in the industry.

In terms of voice-related CA variables, we observed similar trends as in the previous studies [15, 19, 23], specifically feminine-targeted commercials were those most likely to feature singing, female, and children’s voices, and the majority of commercials targeted at mixed audiences featured masculine-only voices. The results of the content analysis were thus in line with previous literature, thereby proving the representativeness of our sample. Moreover, the chi-square tests in Table 1 showed that choices in the casting of voice actors and soundtrack design (e.g., choosing singing over a narrated voice-over) were statistically highly dependent on the gender orientation (i.e., actors in the video) of the commercials, thereby further supporting our understanding of masculine and feminine soundtrack styles as part of distinct gender-based multimodal genres.

Answering *RQ*1, both music-focused perceptual ratings and multimodal emotion ratings appeared to be consistently organized with mixed audience commercials showing in-between values, and masculine and feminine adverts at the two extremes (Tables 2 and 3). Several studies seemed to agree with the notion that boys are much less likely to cross gender lines, which in turn prompts marketers to apply a masculine style to mixed audience commercials [20, 21, 99]. Interviews with children reflect this tendency with regard to color coding, where a “rejection of pink by boys was stronger than any positive liking for pink expressed by the girls” [21]. In our data, this is exemplified by the overrepresentation of masculine voices in mixed audience commercials. Further, using methods of explainable artificial intelligence (XAI) [100], we have shown that mixed audience ads shared more auditory characteristics with masculine-targeted adverts than with feminine-targeted ones. Specifically, we found that the same core set of computationally extracted audio descriptors was selected by machine learning models to distinguish between masculine and feminine-targeted commercials, and between feminine-targeted and mixed audience ads; whereas, the model trained to distinguish between mixed audience and masculine-targeted adverts used a different and larger set of features while achieving the lowest discriminative power.

Excluding the highly collinear “Heavy/Light” scale, the most gender-polarized music descriptors were, by some margin, “High/Low pitch” and “Distorted/Clear”. The observed relationship between pitch level and gender orientation can be easily seen as an iconic reference [101, 102] to the pitch ranges of male and female voices. Similarly, Stronsick et al. [69] provided evidence that pitch level influences the gender ratings of individual instruments, where low pitch levels shifted the ratings in the masculine direction and high pitch levels in the feminine direction. Conversely, distortion was already identified by Machin and Van Leeuwen [103] in their study of music in mobile games as a quality of sound that can become a metaphor for things that can cause roughness in our voice, such as aggressivity. It is no coincidence that roughness was found to be common in male singing from hunting societies that valued assertiveness in boys [103, 104]. A growl-like, harsh sound was also distinctive of male gender-exaggerated voices in ours and previous analyses of gendered toy commercials [19]. Neuroscientific research has also identified auditory roughness as a fundamental feature in both screams and artificial alarm signals, as it engages with subcortical structures that are critical in rapidly *appraising danger* [105]. Another study showed how, even in the absence of emotional or social cues, auditory roughness acts as an innate indication of a threat, prompting defensive responses in the listeners [106]. According to Blumstein et al. [107], distortion elicited similar emotional responses whether applied to musical or vocal sounds, suggesting that the same neurobiological mechanisms play a role in the emotional appraisal of music. In addition, the authors found that a benign visual context (thus, in opposition to the distorted stimulus) could suppress such negative responses, thereby underlining the multimodal nature of music perception (see also Tagg’s notion of codal interference in section 2.2). In the previously mentioned computational analysis, which was conducted on the same dataset as the current study, we found *spectral roughness* to be a distinctive feature of the soundtrack of masculine-targeted commercials [100], where spectral roughness is an estimation of sensory dissonance related to pairs of sinusoids that are close in frequency; namely, the perceptual phenomenon known as auditory roughness. In the sight of these and the current results, we can conclude that nonlinearities in sound (i.e., distortion and roughness) are extensively employed in music for toy adverts as a means to convey danger, aggressivity and, by extension, traditional masculinity; especially seeing how, by comparison, commercials targeted at the feminine audience mostly avoid such sound qualities.

Supporting *H*1_*a*_, the style of masculine-targeted soundtracks was the loudest, heaviest, lowest in pitch, most distorted, least harmonious, least acoustic [74], with the highest tonal density [72], the strongest beats, and the most complex rhythms, while feminine-targeted soundtracks were the softest, lightest, least distorted, most harmonious and acoustic. Similarly, supporting *H*1_*b*_ within the multimodal emotion ratings, masculine-targeted commercials were the most aggressive, and energizing and triumphant, and scored the lowest values on beauty, and calm [72], with feminine-targeted ads displaying the opposite behavior. Traditional gender discourses of masculinity as action-oriented and femininity as passive are echoed throughout the above-mentioned synergistic design choices. Such a traditionalistic view has been consistently found in advertisements aimed at children, not only encoded in music and sound, but also in language, transitions and camera work, setting, interactions, activities, and colors [11, 14–22]. The resulting multimodal genres are in this sense *schema-reinforcing* [65, p. 106-107] fulfilling normative functions. In light of these findings, we posit that gender-based multimodal genres in toy adverts are defined with one another, as it is also directly observable in Figs 3 and 4 (and related interactive maps). It is then no coincidence that, as indicated by surveys in Western countries, men construct their idea of masculinity in constant reference to their understanding of femininity [108]. Here, we reiterated how widespread music-primed gender schemas—which define masculine music as “strong,” “hard,” and “active,” and feminine music as “soft,” “tender,” and “smooth” [41–43]—are likely formed in developmental age [77] precisely through exposure to gender-based multimodal genres that are exemplified by those found in toy TV commercials. In this context, it’s essential to emphasize the music’s ability to affect or arouse the emotions of the listener, since musical depictions of gender can serve as archetypes of gender-appropriate behaviors, function as vehicles for the dissemination of gender-related beliefs, and facilitate the listener’s engagement with distinct gendered subject positions [66, p. 130].

The exploratory factor analysis performed on the music-focused scales found three common latent dimensions across all target audiences. The first of these dimensions attended to acousticness, loudness, tempo, tonal density, and beat strength in a consistent and interpretable manner along what we could call the “Energizing/Soothing” axis. The second one pertained to harmoniousness, melodiousness and degree of distortion, along what we could call the “Harmonious and clear/Dissonant and distorted” axis. The third dimension instead related to the complexity of rhythm, instrumentation and melody, and could be then renamed as the “Complex/Simple” axis. An additional fourth axis relating to pitch height was found for masculine-targeted commercials only, underlining the differences between masculine, mixed, and feminine soundtracks, and further supporting their categorization in distinct multimodal genres.

Finally, answering *RQ*2, the estimated factors scores and the voice-related CA variables were found to explain quite well the multimodal emotions. The first two latent dimensions in particular were found to be easily interpretable. The “Energizing/Soothing” axis was relevant for all emotions for which energy (i.e., arousal) is a defining component [109]. Interestingly, the “Harmonious and clear/Dissonant and distorted” axis was not a significant regressor for any of the emotions within feminine-targeted commercials. Instead, this dimension was ubiquitously significant, and interpretable in terms of both valence and arousal, that is, it influenced emotions in oblique directions within Russell’s circumplex model [109] within masculine and mixed audience adverts, indicating that variation along this axis was more relevant whenever the masculine audience was being addressed. Thus, results from the regression analyses highlighted the importance of the auditory channel in conveying affective content in multimodal communicative events.

### 5.1 Limitations and future directions

This research had some limitations. Within the collected demographics for the aesthetic emotions we noticed that our poll was disproportionately white, with 89% of the participants, while the 2021 UK census put this figure around 82%. Furthermore, having collected commercials intended for the UK market and ratings from UK residents, we could not claim cross-cultural generalizability of the results. Given that many of the commercials in our dataset can be found dubbed in a foreign language, future studies may investigate the extent to which massification and glocalization processes are at play [64].

While raters were instructed to evaluate the music-focused perceptual scales with respect to the music in the background of the soundtracks, it is possible that some bias might have been introduced by the presence of sound effects and speech, although results from our computational study [110] suggested that participants were indeed able to focus on the background music.

Moreover, as previously discussed, modeling the intended emotional profile of the commercials required collecting affective ratings from adults. Future studies may investigate to what extent responses from children (as the primary target audience) would align with those of adults.

Finally, we believe that the proposed hierarchical integrative approach—comprising content analysis, mid-level perceptual features, and multimodal affective responses—has great potential for reuse in different contexts, such as analyzing gender coding in movies and music videos, or even studying media representations of other intersectional factors such as socio-economic status and ethnicity.

### 5.2 Conclusion

This study provided a theoretical foregrounding and empirical proof of the salient role of sound and music in reproducing and perpetuating traditional gender discourse and its power relations within the context of gendered toy marketing. Integrating content analysis with music-focused and multimodal emotion ratings of a set of 606 toy TV commercials spanning over a ten years time frame, we found strong gender polarization in nearly all of the collected variables. Understanding music as multimodal discourse enabled us to frame *music-primed gender schemas* as likely emerging from repeated exposure to masculine and feminine multimodal genres exemplified by those found in toy TV commercials.

Already three decades ago, the renowned music pedagogist—and pioneer in the study of gendered musical practices—Lucy Green [111] wrote that “gendered musical meanings affect our consciousness and experience, not only of music, but through music, of ourselves. Gendered musical meanings participate in the construction of our very notions of masculinity and femininity.” Thus, the authors hope that this work will not only be of interest to scholars but will also encourage policymakers and marketers to break out of this vicious cycle by starting to regulate, among other aspects, the selection and composition of sound and music in advertisements, especially when the targeted audience consists of children in their developmental age.

While much attention in advertising research has been given to the overt sexualization and objectification of visibly non-disabled women, this study underscores the equally insidious impact of enforcing rigid gender stereotypes, particularly in advertisements targeting children. As communicated by the UK Committee of Advertising Practice and the Broadcast Committee of Advertising Practice [8] in December 2020, there is a pressing need for regulatory bodies to shift focus toward the broader implications of (non)conformity to gender norms in advertising messages. Future research-informed policy should address how these stereotypes not only shape individual identity but also limit the diversity of roles and behaviors that children see as attainable.
